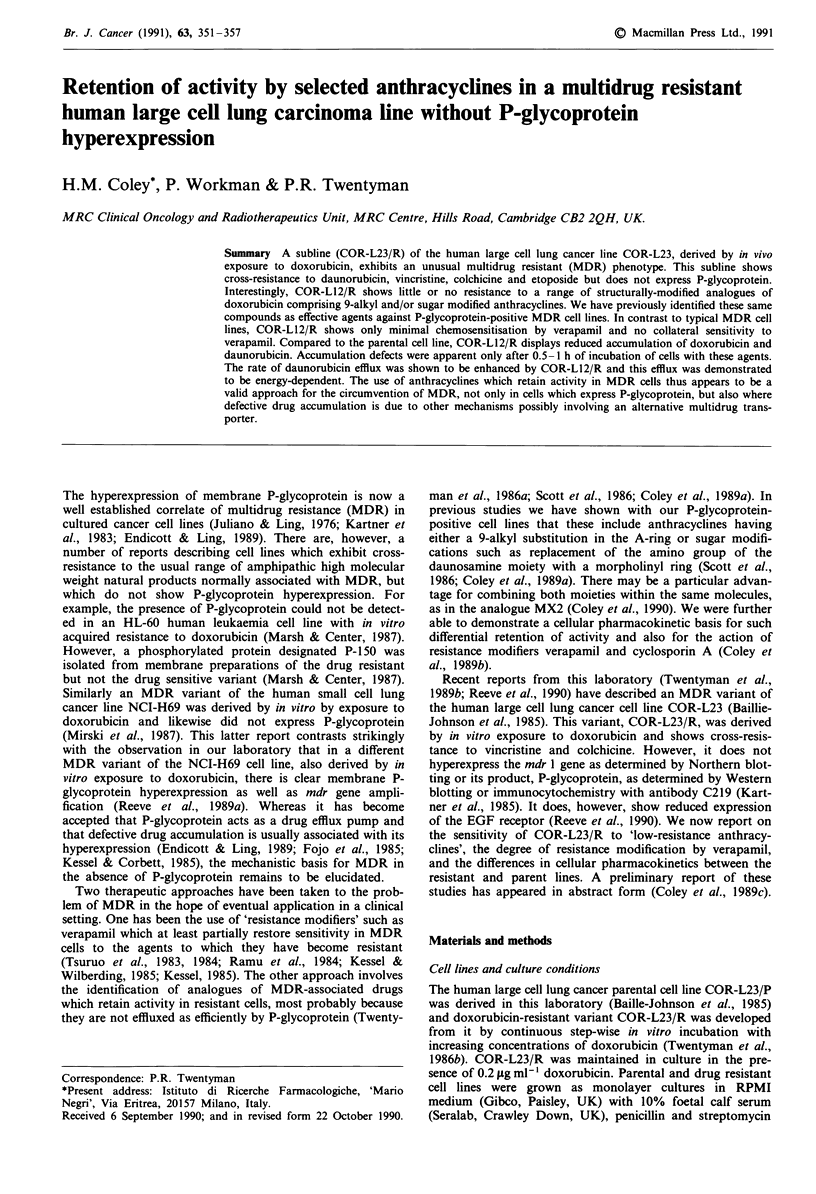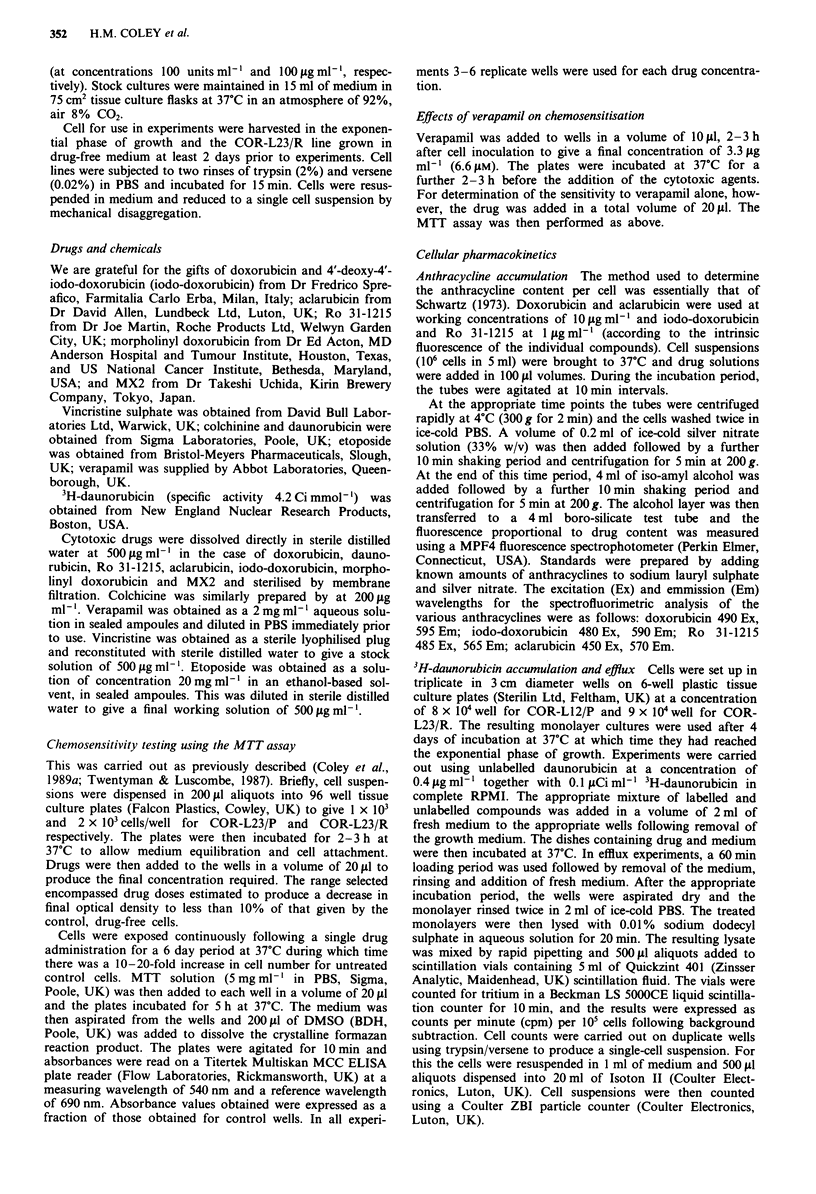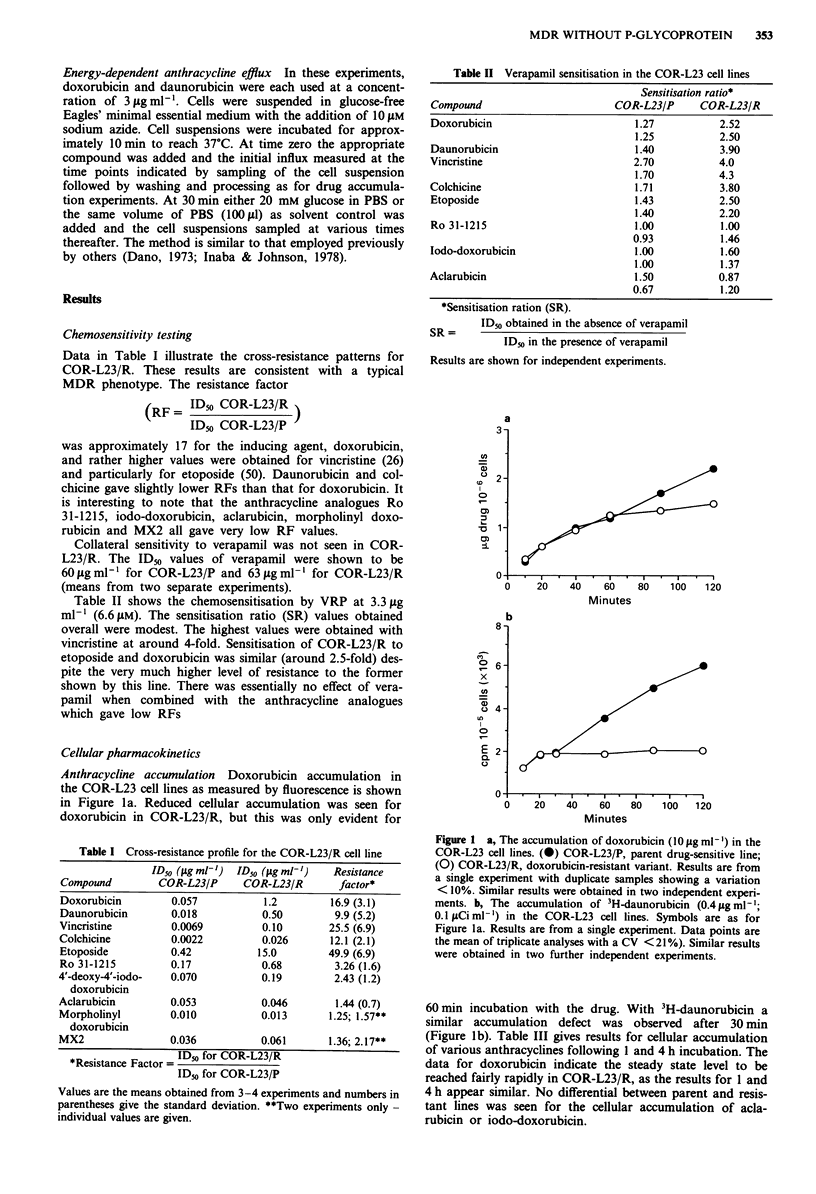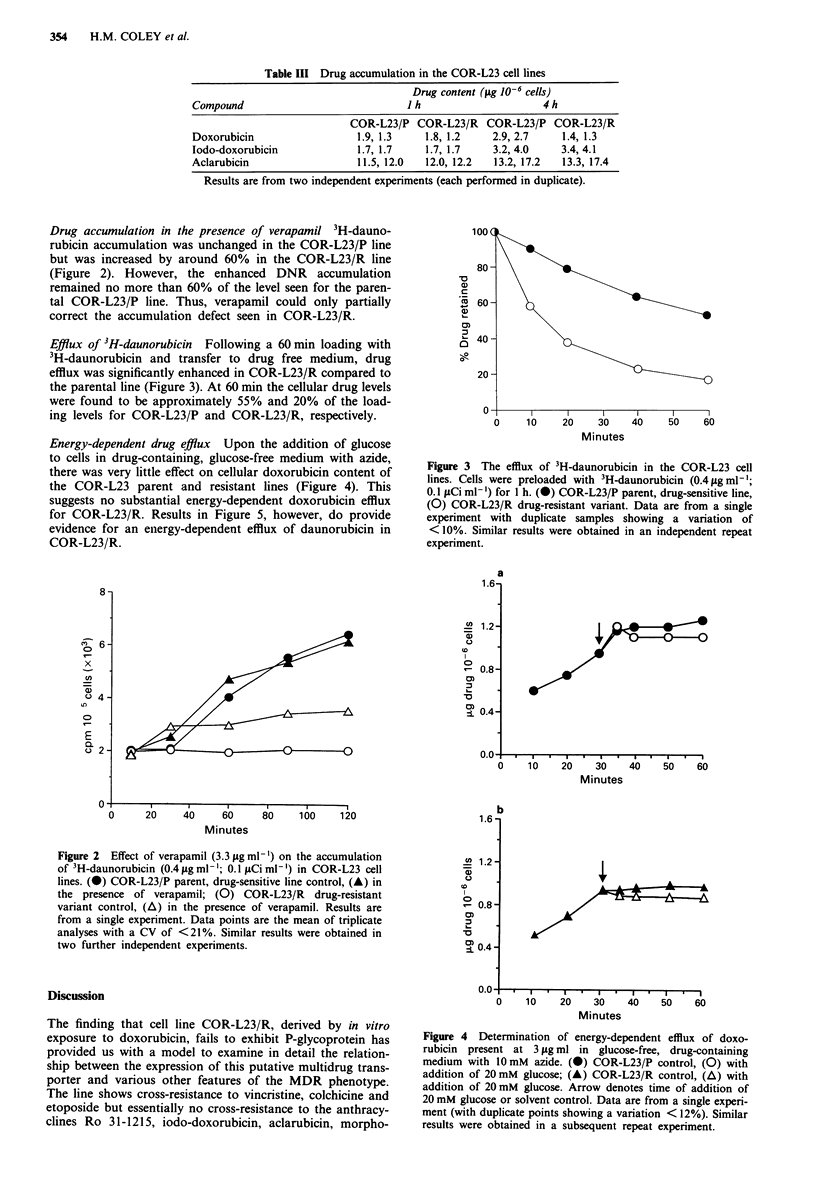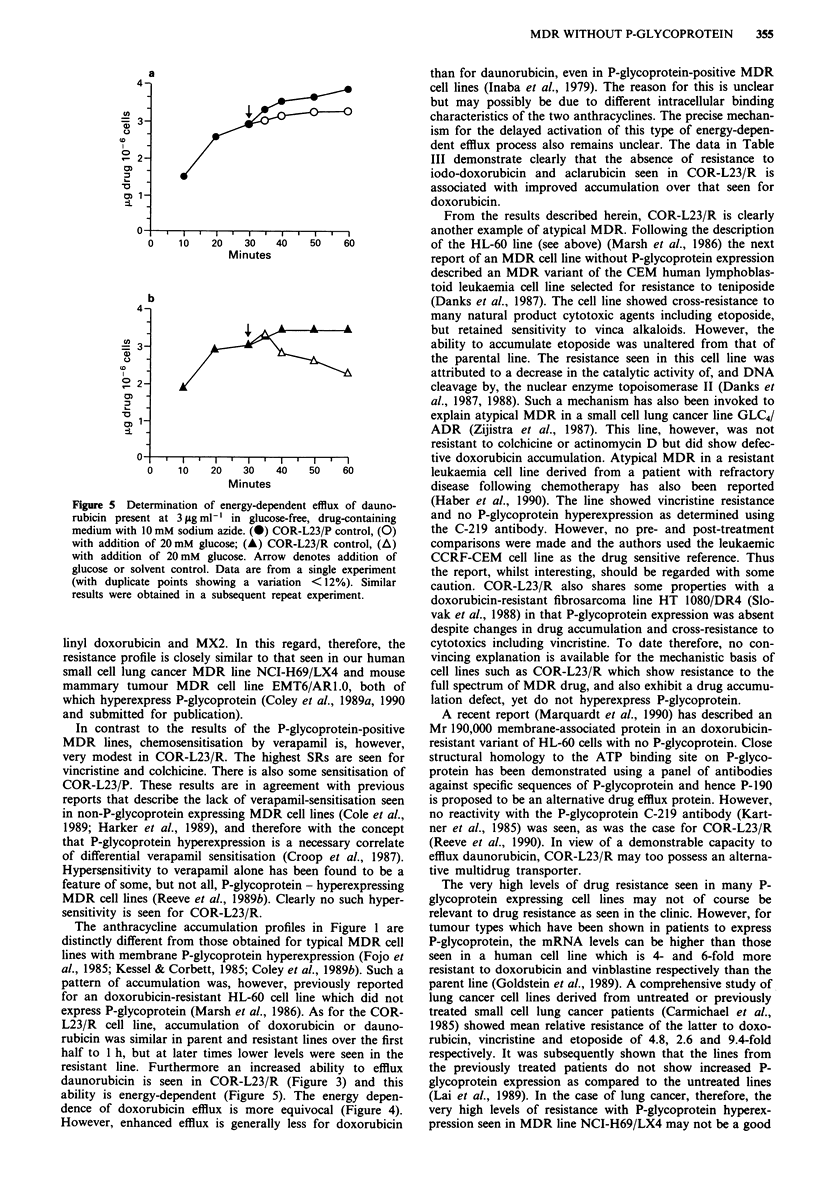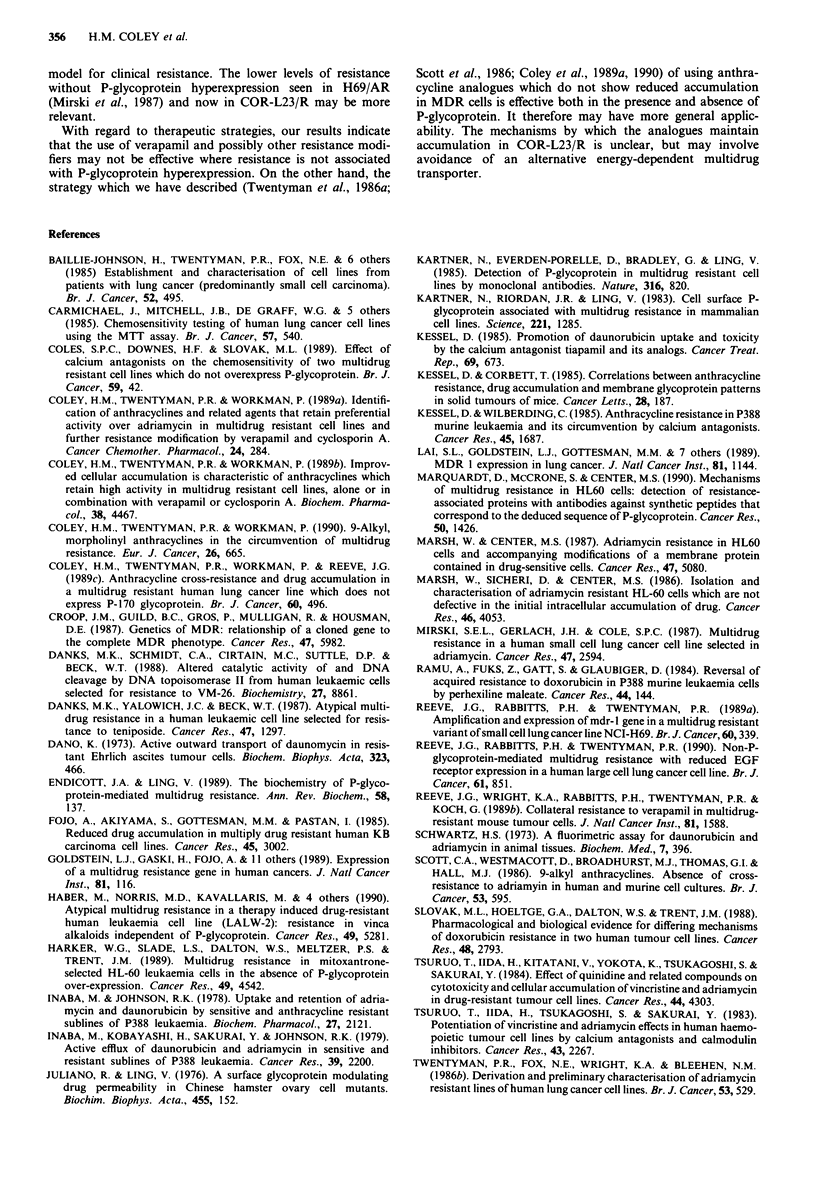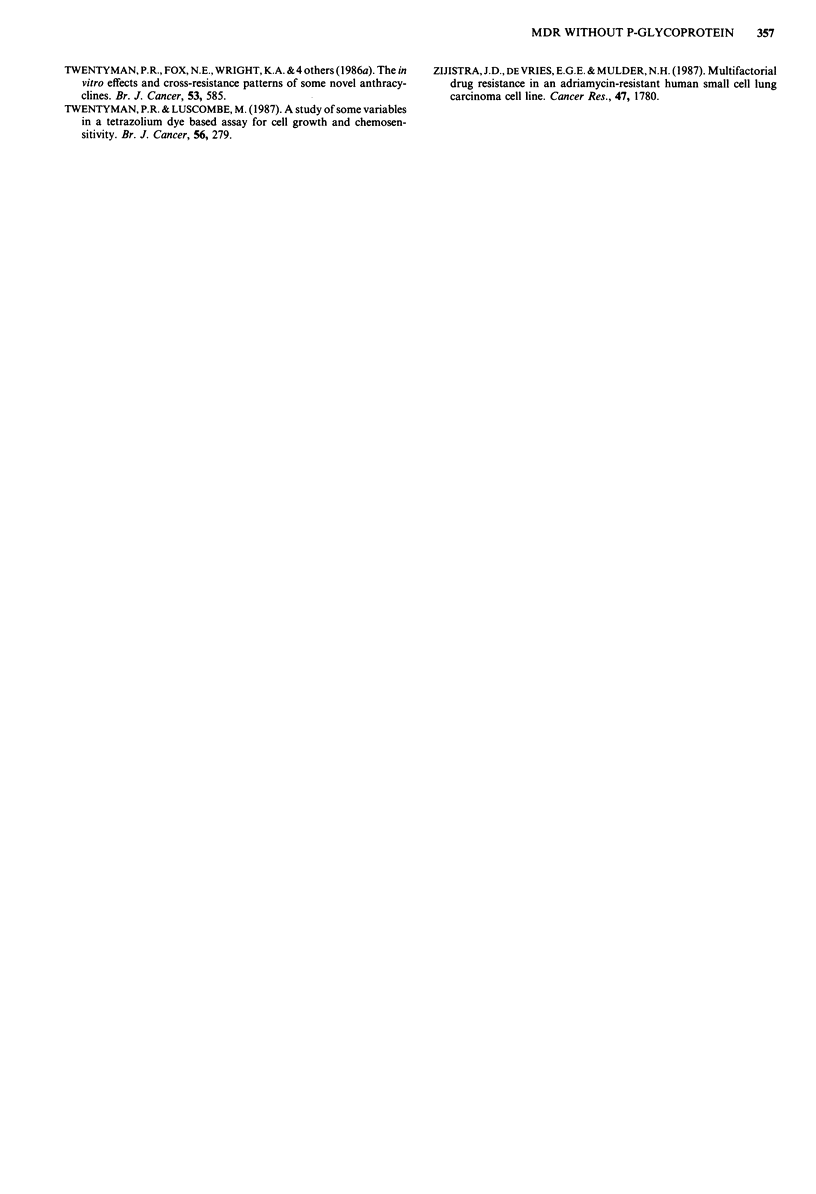# Retention of activity by selected anthracyclines in a multidrug resistant human large cell lung carcinoma line without P-glycoprotein hyperexpression.

**DOI:** 10.1038/bjc.1991.84

**Published:** 1991-03

**Authors:** H. M. Coley, P. Workman, P. R. Twentyman

**Affiliations:** MRC Clinical Oncology and Radiotherapeutics Unit, MRC Centre, Cambridge, UK.

## Abstract

A subline (COR-L23/R) of the human large cell lung line [corrected] COR-L23, derived by in vivo exposure to doxorubicin, exhibits an unusual multidrug resistant (MDR) phenotype. This subline shows cross-resistance to daunorubicin, vincristine, colchicine and etoposide but does not express P-glycoprotein. Interestingly, COR-L23/R [corrected] shows little or no resistance to a range of structurally-modified analogues of doxorubicin comprising 9-alkyl and/or sugar modified anthracyclines. We have previously identified these same compounds as effective agents against P-glycoprotein-positive MDR cell lines. In contrast to typical MDR cell lines, COR-L23/R [corrected] shows only minimal chemosensitisation by verapamil and no collateral sensitivity to verapamil. Compared to the parental cell line, COR-L23/R [corrected] displays reduced accumulation of doxorubicin and daunorubicin. Accumulation defects were apparent only after 0.5-1 h of incubation of cells with these agents. The rate of daunorubicin efflux was shown to be enhanced by COR-L23/R [corrected] and this efflux was demonstrated to be energy-dependent. The use of anthracyclines which retain activity in MDR cells thus appears to be a valid approach for the circumvention of MDR, not only in cells which express P-glycoprotein, but also where defective drug accumulation is due to other mechanisms possibly involving an alternative multidrug transporter.


*Br. J. Cancer* (1991), **63**, 351–357 © Macmillan Press Ltd., 1991

### Retention of activity by selected anthracyclines in a multidrug resistant human large cell lung carcinoma line without P-glycoprotein hyperexpression

H.M. Coley*, P. Workman & P.R. Twentyman

*MRC Clinical Oncology and Radiotherapeutics Unit, MRC Centre, Hills Road, Cambridge CB2 2QH, UK.*

**Summary** A subline (COR-L23/R) of the human large cell lung cancer line COR-L23, derived by *in vivo* exposure to doxorubicin, exhibits an unusual multidrug resistant (MDR) phenotype. This subline shows cross-resistance to daunorubicin, vincristine, colchicine and etoposide but does not express P-glycoprotein. Interestingly, COR-L12/R shows little or no resistance to a range of structurally-modified analogues of doxorubicin comprising 9-alkyl and/or sugar modified anthracyclines. We have previously identified these same compounds as effective agents against P-glycoprotein-positive MDR cell lines. In contrast to typical MDR cell lines, COR-L12/R shows only minimal chemosensitisation by verapamil and no collateral sensitivity to verapamil. Compared to the parental cell line, COR-L12/R displays reduced accumulation of doxorubicin and daunorubicin. Accumulation defects were apparent only after 0.5–1 h of incubation of cells with these agents. The rate of daunorubicin efflux was shown to be enhanced by COR-L12/R and this efflux was demonstrated to be energy-dependent. The use of anthracyclines which retain activity in MDR cells thus appears to be a valid approach for the circumvention of MDR, not only in cells which express P-glycoprotein, but also where defective drug accumulation is due to other mechanisms possibly involving an alternative multidrug transporter.

The hyperexpression of membrane P-glycoprotein is now a well established correlate of multidrug resistance (MDR) in cultured cancer cell lines (Juliano & Ling, 1976; Kartner *et al.*, 1983; Endicott & Ling, 1989). There are, however, a number of reports describing cell lines which exhibit cross-resistance to the usual range of amphipathic high molecular weight natural products normally associated with MDR, but which do not show P-glycoprotein hyperexpression. For example, the presence of P-glycoprotein could not be detected in an HL-60 human leukaemia cell line with *in vitro* acquired resistance to doxorubicin (Marsh & Center, 1987). However, a phosphorylated protein designated P-150 was isolated from membrane preparations of the drug resistant but not the drug sensitive variant (Marsh & Center, 1987). Similarly an MDR variant of the human small cell lung cancer line NCI-H69 was derived by *in vitro* by exposure to doxorubicin and likewise did not express P-glycoprotein (Mirski *et al.*, 1987). This latter report contrasts strikingly with the observation in our laboratory that in a different MDR variant of the NCI-H69 cell line, also derived by *in vitro* exposure to doxorubicin, there is clear membrane P-glycoprotein hyperexpression as well as *mdr* gene amplification (Reeve *et al.*, 1989*a*). Whereas it has become accepted that P-glycoprotein acts as a drug efflux pump and that defective drug accumulation is usually associated with its hyperexpression (Endicott & Ling, 1989; Fojo *et al.*, 1985; Kessel & Corbett, 1985), the mechanistic basis for MDR in the absence of P-glycoprotein remains to be elucidated.

Two therapeutic approaches have been taken to the problem of MDR in the hope of eventual application in a clinical setting. One has been the use of 'resistance modifiers' such as verapamil which at least partially restore sensitivity in MDR cells to the agents to which they have become resistant (Tsuruo *et al.*, 1983, 1984; Ramu *et al.*, 1984; Kessel & Wilberding, 1985; Kessel, 1985). The other approach involves the identification of analogues of MDR-associated drugs which retain activity in resistant cells, most probably because they are not effluxed as efficiently by P-glycoprotein (Twentyman *et al.*, 1986*a*; Scott *et al.*, 1986; Coley *et al.*, 1989*a*). In previous studies we have shown with our P-glycoprotein-positive cell lines that these include anthracyclines having either a 9-alkyl substitution in the A-ring or sugar modifications such as replacement of the amino group of the daunosamine moiety with a morpholinyl ring (Scott *et al.*, 1986; Coley *et al.*, 1989*a*). There may be a particular advantage for combining both moieties within the same molecules, as in the analogue MX2 (Coley *et al.*, 1990). We were further able to demonstrate a cellular pharmacokinetic basis for such differential retention of activity and also for the action of resistance modifiers verapamil and cyclosporin A (Coley *et al.*, 1989*b*).

Recent reports from this laboratory (Twentyman *et al.*, 1989*b*; Reeve *et al.*, 1990) have described an MDR variant of the human large cell lung cancer cell line COR-L23 (Baillie-Johnson *et al.*, 1985). This variant, COR-L23/R, was derived by *in vitro* exposure to doxorubicin and shows cross-resistance to vincristine and colchicine. However, it does not hyperexpress the *mdr* 1 gene as determined by Northern blotting or its product, P-glycoprotein, as determined by Western blotting or immunocytochemistry with antibody C219 (Kartner *et al.*, 1985). It does, however, show reduced expression of the EGF receptor (Reeve *et al.*, 1990). We now report on the sensitivity of COR-L23/R to 'low-resistance anthracyclines', the degree of resistance modification by verapamil, and the differences in cellular pharmacokinetics between the resistant and parent lines. A preliminary report of these studies has appeared in abstract form (Coley *et al.*, 1989*c*).

### Materials and methods

### *Cell lines and culture conditions*

The human large cell lung cancer parental cell line COR-L23/P was derived in this laboratory (Baille-Johnson *et al.*, 1985) and doxorubicin-resistant variant COR-L23/R was developed from it by continuous step-wise *in vitro* incubation with increasing concentrations of doxorubicin (Twentyman *et al.*, 1986*b*). COR-L23/R was maintained in culture in the presence of 0.2 $\mu g\ ml^{-1}$ doxorubicin. Parental and drug resistant cell lines were grown as monolayer cultures in RPMI medium (Gibco, Paisley, UK) with 10% foetal calf serum (Seralab, Crawley Down, UK), penicillin and streptomycin

Correspondence: P.R. Twentyman
*Present address: Istituto di Ricerche Farmacologiche, 'Mario Negri', Via Eritrea, 20157 Milano, Italy.
Received 6 September 1990; and in revised form 22 October 1990.

(at concentrations 100 units $ml^{-1}$ and 100 $\mu g$ $ml^{-1}$, respectively). Stock cultures were maintained in 15 ml of medium in 75 $cm^2$ tissue culture flasks at 37°C in an atmosphere of 92%, air 8% $CO_2$.

Cell for use in experiments were harvested in the exponential phase of growth and the COR-L23/R line grown in drug-free medium at least 2 days prior to experiments. Cell lines were subjected to two rinses of trypsin (2%) and versene (0.02%) in PBS and incubated for 15 min. Cells were resuspended in medium and reduced to a single cell suspension by mechanical disaggregation.

*Drugs and chemicals*

We are grateful for the gifts of doxorubicin and 4′-deoxy-4′-iodo-doxorubicin (iodo-doxorubicin) from Dr Fredrico Spreafico, Farmitalia Carlo Erba, Milan, Italy; aclarubicin from Dr David Allen, Lundbeck Ltd, Luton, UK; Ro 31-1215 from Dr Joe Martin, Roche Products Ltd, Welwyn Garden City, UK; morpholinyl doxorubicin from Dr Ed Acton, MD Anderson Hospital and Tumour Institute, Houston, Texas, and US National Cancer Institute, Bethesda, Maryland, USA; and MX2 from Dr Takeshi Uchida, Kirin Brewery Company, Tokyo, Japan.

Vincristine sulphate was obtained from David Bull Laboratories Ltd, Warwick, UK; colchinine and daunorubicin were obtained from Sigma Laboratories, Poole, UK; etoposide was obtained from Bristol-Meyers Pharmaceuticals, Slough, UK; verapamil was supplied by Abbot Laboratories, Queenborough, UK.

$^3$H-daunorubicin (specific activity 4.2 Ci $mmol^{-1}$) was obtained from New England Nuclear Research Products, Boston, USA.

Cytotoxic drugs were dissolved directly in sterile distilled water at 500 $\mu g$ $ml^{-1}$ in the case of doxorubicin, daunorubicin, Ro 31-1215, aclarubicin, iodo-doxorubicin, morpholinyl doxorubicin and MX2 and sterilised by membrane filtration. Colchicine was similarly prepared by at 200 $\mu g$ $ml^{-1}$. Verapamil was obtained as a 2 mg $ml^{-1}$ aqueous solution in sealed ampoules and diluted in PBS immediately prior to use. Vincristine was obtained as a sterile lyophilised plug and reconstituted with sterile distilled water to give a stock solution of 500 $\mu g$ $ml^{-1}$. Etoposide was obtained as a solution of concentration 20 mg $ml^{-1}$ in an ethanol-based solvent, in sealed ampoules. This was diluted in sterile distilled water to give a final working solution of 500 $\mu g$ $ml^{-1}$.

*Chemosensitivity testing using the MTT assay*

This was carried out as previously described (Coley *et al.*, 1989*a*; Twentyman & Luscombe, 1987). Briefly, cell suspensions were dispensed in 200 $\mu l$ aliquots into 96 well tissue culture plates (Falcon Plastics, Cowley, UK) to give $1 \times 10^3$ and $2 \times 10^3$ cells/well for COR-L23/P and COR-L23/R respectively. The plates were then incubated for 2–3 h at 37°C to allow medium equilibration and cell attachment. Drugs were then added to the wells in a volume of 20 $\mu l$ to produce the final concentration required. The range selected encompassed drug doses estimated to produce a decrease in final optical density to less than 10% of that given by the control, drug-free cells.

Cells were exposed continuously following a single drug administration for a 6 day period at 37°C during which time there was a 10–20-fold increase in cell number for untreated control cells. MTT solution (5 mg $ml^{-1}$ in PBS, Sigma, Poole, UK) was then added to each well in a volume of 20 $\mu l$ and the plates incubated for 5 h at 37°C. The medium was then aspirated from the wells and 200 $\mu l$ of DMSO (BDH, Poole, UK) was added to dissolve the crystalline formazan reaction product. The plates were agitated for 10 min and absorbances were read on a Titertek Multiskan MCC ELISA plate reader (Flow Laboratories, Rickmansworth, UK) at a measuring wavelength of 540 nm and a reference wavelength of 690 nm. Absorbance values obtained were expressed as a fraction of those obtained for control wells. In all experiments 3–6 replicate wells were used for each drug concentration.

*Effects of verapamil on chemosensitisation*

Verapamil was added to wells in a volume of 10 $\mu l$, 2–3 h after cell inoculation to give a final concentration of 3.3 $\mu g$ $ml^{-1}$ (6.6 $\mu M$). The plates were incubated at 37°C for a further 2–3 h before the addition of the cytotoxic agents. For determination of the sensitivity to verapamil alone, however, the drug was added in a total volume of 20 $\mu l$. The MTT assay was then performed as above.

*Cellular pharmacokinetics*

*Anthracycline accumulation* The method used to determine the anthracycline content per cell was essentially that of Schwartz (1973). Doxorubicin and aclarubicin were used at working concentrations of 10 $\mu g$ $ml^{-1}$ and iodo-doxorubicin and Ro 31-1215 at 1 $\mu g$ $ml^{-1}$ (according to the intrinsic fluorescence of the individual compounds). Cell suspensions ($10^6$ cells in 5 ml) were brought to 37°C and drug solutions were added in 100 $\mu l$ volumes. During the incubation period, the tubes were agitated at 10 min intervals.

At the appropriate time points the tubes were centrifuged rapidly at 4°C (300 *g* for 2 min) and the cells washed twice in ice-cold PBS. A volume of 0.2 ml of ice-cold silver nitrate solution (33% w/v) was then added followed by a further 10 min shaking period and centrifugation for 5 min at 200 *g*. At the end of this time period, 4 ml of iso-amyl alcohol was added followed by a further 10 min shaking period and centrifugation for 5 min at 200 *g*. The alcohol layer was then transferred to a 4 ml boro-silicate test tube and the fluorescence proportional to drug content was measured using a MPF4 fluorescence spectrophotometer (Perkin Elmer, Connecticut, USA). Standards were prepared by adding known amounts of anthracyclines to sodium lauryl sulphate and silver nitrate. The excitation (Ex) and emmission (Em) wavelengths for the spectrofluorimetric analysis of the various anthracyclines were as follows: doxorubicin 490 Ex, 595 Em; iodo-doxorubicin 480 Ex, 590 Em; Ro 31-1215 485 Ex, 565 Em; aclarubicin 450 Ex, 570 Em.

*$^3$H-daunorubicin accumulation and efflux* Cells were set up in triplicate in 3 cm diameter wells on 6-well plastic tissue culture plates (Sterilin Ltd, Feltham, UK) at a concentration of $8 \times 10^4$ well for COR-L12/P and $9 \times 10^4$ well for COR-L23/R. The resulting monolayer cultures were used after 4 days of incubation at 37°C at which time they had reached the exponential phase of growth. Experiments were carried out using unlabelled daunorubicin at a concentration of 0.4 $\mu g$ $ml^{-1}$ together with 0.1 $\mu Ci$ $ml^{-1}$ $^3$H-daunorubicin in complete RPMI. The appropriate mixture of labelled and unlabelled compounds was added in a volume of 2 ml of fresh medium to the appropriate wells following removal of the growth medium. The dishes containing drug and medium were then incubated at 37°C. In efflux experiments, a 60 min loading period was used followed by removal of the medium, rinsing and addition of fresh medium. After the appropriate incubation period, the wells were aspirated dry and the monolayer rinsed twice in 2 ml of ice-cold PBS. The treated monolayers were then lysed with 0.01% sodium dodecyl sulphate in aqueous solution for 20 min. The resulting lysate was mixed by rapid pipetting and 500 $\mu l$ aliquots added to scintillation vials containing 5 ml of Quickzint 401 (Zinsser Analytic, Maidenhead, UK) scintillation fluid. The vials were counted for tritium in a Beckman LS 5000CE liquid scintillation counter for 10 min, and the results were expressed as counts per minute (cpm) per $10^5$ cells following background subtraction. Cell counts were carried out on duplicate wells using trypsin/versene to produce a single-cell suspension. For this the cells were resuspended in 1 ml of medium and 500 $\mu l$ aliquots dispensed into 20 ml of Isoton II (Coulter Electronics, Luton, UK). Cell suspensions were then counted using a Coulter ZBI particle counter (Coulter Electronics, Luton, UK).

*Energy-dependent anthracycline efflux* In these experiments, doxorubicin and daunorubicin were each used at a concentration of 3 μg ml$^{-1}$. Cells were suspended in glucose-free Eagles' minimal essential medium with the addition of 10 μM sodium azide. Cell suspensions were incubated for approximately 10 min to reach 37°C. At time zero the appropriate compound was added and the initial influx measured at the time points indicated by sampling of the cell suspension followed by washing and processing as for drug accumulation experiments. At 30 min either 20 mM glucose in PBS or the same volume of PBS (100 μl) as solvent control was added and the cell suspensions sampled at various times thereafter. The method is similar to that employed previously by others (Dano, 1973; Inaba & Johnson, 1978).

### Results

### *Chemosensitivity testing*

Data in Table I illustrate the cross-resistance patterns for COR-L23/R. These results are consistent with a typical MDR phenotype. The resistance factor

$$\left(RF = \frac{ID_{50}\ \text{COR-L23/R}}{ID_{50}\ \text{COR-L23/P}}\right)$$

was approximately 17 for the inducing agent, doxorubicin, and rather higher values were obtained for vincristine (26) and particularly for etoposide (50). Daunorubicin and colchicine gave slightly lower RFs than that for doxorubicin. It is interesting to note that the anthracycline analogues Ro 31-1215, iodo-doxorubicin, aclarubicin, morpholinyl doxorubicin and MX2 all gave very low RF values.

Collateral sensitivity to verapamil was not seen in COR-L23/R. The $ID_{50}$ values of verapamil were shown to be 60 μg ml$^{-1}$ for COR-L23/P and 63 μg ml$^{-1}$ for COR-L23/R (means from two separate experiments).

Table II shows the chemosensitisation by VRP at 3.3 μg ml$^{-1}$ (6.6 μM). The sensitisation ratio (SR) values obtained overall were modest. The highest values were obtained with vincristine at around 4-fold. Sensitisation of COR-L23/R to etoposide and doxorubicin was similar (around 2.5-fold) despite the very much higher level of resistance to the former shown by this line. There was essentially no effect of verapamil when combined with the anthracycline analogues which gave low RFs

### *Cellular pharmacokinetics*

*Anthracycline accumulation* Doxorubicin accumulation in the COR-L23 cell lines as measured by fluorescence is shown in Figure 1a. Reduced cellular accumulation was seen for doxorubicin in COR-L23/R, but this was only evident for 60 min incubation with the drug. With $^3$H-daunorubicin a similar accumulation defect was observed after 30 min (Figure 1b). Table III gives results for cellular accumulation of various anthracyclines following 1 and 4 h incubation. The data for doxorubicin indicate the steady state level to be reached fairly rapidly in COR-L23/R, as the results for 1 and 4 h appear similar. No differential between parent and resistant lines was seen for the cellular accumulation of aclarubicin or iodo-doxorubicin.

**Table I** Cross-resistance profile for the COR-L23/R cell line

| *Compound* | *$ID_{50}$ (μg ml$^{-1}$) COR-L23/P* | *$ID_{50}$ (μg ml$^{-1}$) COR-L23/R* | *Resistance factor** |
|---|---|---|---|
| Doxorubicin | 0.057 | 1.2 | 16.9 (3.1) |
| Daunorubicin | 0.018 | 0.50 | 9.9 (5.2) |
| Vincristine | 0.0069 | 0.10 | 25.5 (6.9) |
| Colchicine | 0.0022 | 0.026 | 12.1 (2.1) |
| Etoposide | 0.42 | 15.0 | 49.9 (6.9) |
| Ro 31-1215 | 0.17 | 0.68 | 3.26 (1.6) |
| 4'-deoxy-4'-iodo-doxorubicin | 0.070 | 0.19 | 2.43 (1.2) |
| Aclarubicin | 0.053 | 0.046 | 1.44 (0.7) |
| Morpholinyl doxorubicin | 0.010 | 0.013 | 1.25; 1.57** |
| MX2 | 0.036 | 0.061 | 1.36; 2.17** |

$$*\text{Resistance Factor} = \frac{ID_{50}\ \text{for COR-L23/R}}{ID_{50}\ \text{for COR-L23/P}}$$

Values are the means obtained from 3–4 experiments and numbers in parentheses give the standard deviation. **Two experiments only – individual values are given.

**Table II** Verapamil sensitisation in the COR-L23 cell lines

| *Compound* | *Sensitisation ratio** *COR-L23/P* | *COR-L23/R* |
|---|---|---|
| Doxorubicin | 1.27 | 2.52 |
| | 1.25 | 2.50 |
| Daunorubicin | 1.40 | 3.90 |
| Vincristine | 2.70 | 4.0 |
| | 1.70 | 4.3 |
| Colchicine | 1.71 | 3.80 |
| Etoposide | 1.43 | 2.50 |
| | 1.40 | 2.20 |
| Ro 31-1215 | 1.00 | 1.00 |
| | 0.93 | 1.46 |
| Iodo-doxorubicin | 1.00 | 1.60 |
| | 1.00 | 1.37 |
| Aclarubicin | 1.50 | 0.87 |
| | 0.67 | 1.20 |

*Sensitisation ration (SR).

$$SR = \frac{ID_{50}\ \text{obtained in the absence of verapamil}}{ID_{50}\ \text{in the presence of verapamil}}$$

Results are shown for independent experiments.

**Figure 1** **a**, The accumulation of doxorubicin (10 μg ml$^{-1}$) in the COR-L23 cell lines. (●) COR-L23/P, parent drug-sensitive line; (○) COR-L23/R, doxorubicin-resistant variant. Results are from a single experiment with duplicate samples showing a variation <10%. Similar results were obtained in two independent experiments. **b**, The accumulation of $^3$H-daunorubicin (0.4 μg ml$^{-1}$; 0.1 μCi ml$^{-1}$) in the COR-L23 cell lines. Symbols are as for Figure 1a. Results are from a single experiment. Data points are the mean of triplicate analyses with a CV <21%). Similar results were obtained in two further independent experiments.

**Table III** Drug accumulation in the COR-L23 cell lines

| Compound | Drug content ($\mu g\ 10^{-6}$ cells) 1 h | | 4 h | |
|---|---|---|---|---|
| | COR-L23/P | COR-L23/R | COR-L23/P | COR-L23/R |
| Doxorubicin | 1.9, 1.3 | 1.8, 1.2 | 2.9, 2.7 | 1.4, 1.3 |
| Iodo-doxorubicin | 1.7, 1.7 | 1.7, 1.7 | 3.2, 4.0 | 3.4, 4.1 |
| Aclarubicin | 11.5, 12.0 | 12.0, 12.2 | 13.2, 17.2 | 13.3, 17.4 |

Results are from two independent experiments (each performed in duplicate).

*Drug accumulation in the presence of verapamil* $^{3}$H-daunorubicin accumulation was unchanged in the COR-L23/P line but was increased by around 60% in the COR-L23/R line (Figure 2). However, the enhanced DNR accumulation remained no more than 60% of the level seen for the parental COR-L23/P line. Thus, verapamil could only partially correct the accumulation defect seen in COR-L23/R.

*Efflux of $^{3}$H-daunorubicin* Following a 60 min loading with $^{3}$H-daunorubicin and transfer to drug free medium, drug efflux was significantly enhanced in COR-L23/R compared to the parental line (Figure 3). At 60 min the cellular drug levels were found to be approximately 55% and 20% of the loading levels for COR-L23/P and COR-L23/R, respectively.

*Energy-dependent drug efflux* Upon the addition of glucose to cells in drug-containing, glucose-free medium with azide, there was very little effect on cellular doxorubicin content of the COR-L23 parent and resistant lines (Figure 4). This suggests no substantial energy-dependent doxorubicin efflux for COR-L23/R. Results in Figure 5, however, do provide evidence for an energy-dependent efflux of daunorubicin in COR-L23/R.

**Figure 2** Effect of verapamil (3.3 $\mu g\ ml^{-1}$) on the accumulation of $^{3}$H-daunorubicin (0.4 $\mu g\ ml^{-1}$; 0.1 $\mu Ci\ ml^{-1}$) in COR-L23 cell lines. (●) COR-L23/P parent, drug-sensitive line control, (▲) in the presence of verapamil; (O) COR-L23/R drug-resistant variant control, (Δ) in the presence of verapamil. Results are from a single experiment. Data points are the mean of triplicate analyses with a CV of <21%. Similar results were obtained in two further independent experiments.

### Discussion

The finding that cell line COR-L23/R, derived by *in vitro* exposure to doxorubicin, fails to exhibit P-glycoprotein has provided us with a model to examine in detail the relationship between the expression of this putative multidrug transporter and various other features of the MDR phenotype. The line shows cross-resistance to vincristine, colchicine and etoposide but essentially no cross-resistance to the anthracyclines Ro 31-1215, iodo-doxorubicin, aclarubicin, morpho-

**Figure 3** The efflux of $^{3}$H-daunorubicin in the COR-L23 cell lines. Cells were preloaded with $^{3}$H-daunorubicin (0.4 $\mu g\ ml^{-1}$; 0.1 $\mu Ci\ ml^{-1}$) for 1 h. (●) COR-L23/P parent, drug-sensitive line, (O) COR-L23/R drug-resistant variant. Data are from a single experiment with duplicate samples showing a variation of <10%. Similar results were obtained in an independent repeat experiment.

**Figure 4** Determination of energy-dependent efflux of doxorubicin present at 3 $\mu g$ ml in glucose-free, drug-containing medium with 10 mM azide. (●) COR-L23/P control, (O) with addition of 20 mM glucose; (▲) COR-L23/R control, (Δ) with addition of 20 mM glucose. Arrow denotes time of addition of 20 mM glucose or solvent control. Data are from a single experiment (with duplicate points showing a variation <12%). Similar results were obtained in a subsequent repeat experiment.

**Figure 5** Determination of energy-dependent efflux of daunorubicin present at 3 µg ml⁻¹ in glucose-free, drug-containing medium with 10 mM sodium azide. (●) COR-L23/P control, (○) with addition of 20 mM glucose; (▲) COR-L23/R control, (△) with addition of 20 mM glucose. Arrow denotes addition of glucose or solvent control. Data are from a single experiment (with duplicate points showing a variation <12%). Similar results were obtained in a subsequent repeat experiment.

linyl doxorubicin and MX2. In this regard, therefore, the resistance profile is closely similar to that seen in our human small cell lung cancer MDR line NCI-H69/LX4 and mouse mammary tumour MDR cell line EMT6/AR1.0, both of which hyperexpress P-glycoprotein (Coley *et al.*, 1989*a*, 1990 and submitted for publication).

In contrast to the results of the P-glycoprotein-positive MDR lines, chemosensitisation by verapamil is, however, very modest in COR-L23/R. The highest SRs are seen for vincristine and colchicine. There is also some sensitisation of COR-L23/P. These results are in agreement with previous reports that describe the lack of verapamil-sensitisation seen in non-P-glycoprotein expressing MDR cell lines (Cole *et al.*, 1989; Harker *et al.*, 1989), and therefore with the concept that P-glycoprotein hyperexpression is a necessary correlate of differential verapamil sensitisation (Croop *et al.*, 1987). Hypersensitivity to verapamil alone has been found to be a feature of some, but not all, P-glycoprotein – hyperexpressing MDR cell lines (Reeve *et al.*, 1989*b*). Clearly no such hypersensitivity is seen for COR-L23/R.

The anthracycline accumulation profiles in Figure 1 are distinctly different from those obtained for typical MDR cell lines with membrane P-glycoprotein hyperexpression (Fojo *et al.*, 1985; Kessel & Corbett, 1985; Coley *et al.*, 1989*b*). Such a pattern of accumulation was, however, previously reported for an doxorubicin-resistant HL-60 cell line which did not express P-glycoprotein (Marsh *et al.*, 1986). As for the COR-L23/R cell line, accumulation of doxorubicin or daunorubicin was similar in parent and resistant lines over the first half to 1 h, but at later times lower levels were seen in the resistant line. Furthermore an increased ability to efflux daunorubicin is seen in COR-L23/R (Figure 3) and this ability is energy-dependent (Figure 5). The energy dependence of doxorubicin efflux is more equivocal (Figure 4). However, enhanced efflux is generally less for doxorubicin than for daunorubicin, even in P-glycoprotein-positive MDR cell lines (Inaba *et al.*, 1979). The reason for this is unclear but may possibly be due to different intracellular binding characteristics of the two anthracyclines. The precise mechanism for the delayed activation of this type of energy-dependent efflux process also remains unclear. The data in Table III demonstrate clearly that the absence of resistance to iodo-doxorubicin and aclarubicin seen in COR-L23/R is associated with improved accumulation over that seen for doxorubicin.

From the results described herein, COR-L23/R is clearly another example of atypical MDR. Following the description of the HL-60 line (see above) (Marsh *et al.*, 1986) the next report of an MDR cell line without P-glycoprotein expression described an MDR variant of the CEM human lymphoblastoid leukaemia cell line selected for resistance to teniposide (Danks *et al.*, 1987). The cell line showed cross-resistance to many natural product cytotoxic agents including etoposide, but retained sensitivity to vinca alkaloids. However, the ability to accumulate etoposide was unaltered from that of the parental line. The resistance seen in this cell line was attributed to a decrease in the catalytic activity of, and DNA cleavage by, the nuclear enzyme topoisomerase II (Danks *et al.*, 1987, 1988). Such a mechanism has also been invoked to explain atypical MDR in a small cell lung cancer line $GLC_4$/ADR (Zijistra *et al.*, 1987). This line, however, was not resistant to colchicine or actinomycin D but did show defective doxorubicin accumulation. Atypical MDR in a resistant leukaemia cell line derived from a patient with refractory disease following chemotherapy has also been reported (Haber *et al.*, 1990). The line showed vincristine resistance and no P-glycoprotein hyperexpression as determined using the C-219 antibody. However, no pre- and post-treatment comparisons were made and the authors used the leukaemic CCRF-CEM cell line as the drug sensitive reference. Thus the report, whilst interesting, should be regarded with some caution. COR-L23/R also shares some properties with a doxorubicin-resistant fibrosarcoma line HT 1080/DR4 (Slovak *et al.*, 1988) in that P-glycoprotein expression was absent despite changes in drug accumulation and cross-resistance to cytotoxics including vincristine. To date therefore, no convincing explanation is available for the mechanistic basis of cell lines such as COR-L23/R which show resistance to the full spectrum of MDR drug, and also exhibit a drug accumulation defect, yet do not hyperexpress P-glycoprotein.

A recent report (Marquardt *et al.*, 1990) has described an Mr 190,000 membrane-associated protein in an doxorubicin-resistant variant of HL-60 cells with no P-glycoprotein. Close structural homology to the ATP binding site on P-glycoprotein has been demonstrated using a panel of antibodies against specific sequences of P-glycoprotein and hence P-190 is proposed to be an alternative drug efflux protein. However, no reactivity with the P-glycoprotein C-219 antibody (Kartner *et al.*, 1985) was seen, as was the case for COR-L23/R (Reeve *et al.*, 1990). In view of a demonstrable capacity to efflux daunorubicin, COR-L23/R may too possess an alternative multidrug transporter.

The very high levels of drug resistance seen in many P-glycoprotein expressing cell lines may not of course be relevant to drug resistance as seen in the clinic. However, for tumour types which have been shown in patients to express P-glycoprotein, the mRNA levels can be higher than those seen in a human cell line which is 4- and 6-fold more resistant to doxorubicin and vinblastine respectively than the parent line (Goldstein *et al.*, 1989). A comprehensive study of lung cancer cell lines derived from untreated or previously treated small cell lung cancer patients (Carmichael *et al.*, 1985) showed mean relative resistance of the latter to doxorubicin, vincristine and etoposide of 4.8, 2.6 and 9.4-fold respectively. It was subsequently shown that the lines from the previously treated patients do not show increased P-glycoprotein expression as compared to the untreated lines (Lai *et al.*, 1989). In the case of lung cancer, therefore, the very high levels of resistance with P-glycoprotein hyperexpression seen in MDR line NCI-H69/LX4 may not be a good model for clinical resistance. The lower levels of resistance without P-glycoprotein hyperexpression seen in H69/AR (Mirski *et al.*, 1987) and now in COR-L23/R may be more relevant.

With regard to therapeutic strategies, our results indicate that the use of verapamil and possibly other resistance modifiers may not be effective where resistance is not associated with P-glycoprotein hyperexpression. On the other hand, the strategy which we have described (Twentyman *et al.*, 1986*a*; Scott *et al.*, 1986; Coley *et al.*, 1989*a*, 1990) of using anthracycline analogues which do not show reduced accumulation in MDR cells is effective both in the presence and absence of P-glycoprotein. It therefore may have more general applicability. The mechanisms by which the analogues maintain accumulation in COR-L23/R is unclear, but may involve avoidance of an alternative energy-dependent multidrug transporter.

### References

BAILLIE-JOHNSON, H., TWENTYMAN, P.R., FOX, N.E. & 6 others (1985) Establishment and characterisation of cell lines from patients with lung cancer (predominantly small cell carcinoma). *Br. J. Cancer*, **52**, 495.

CARMICHAEL, J., MITCHELL, J.B., DE GRAFF, W.G. & 5 others (1985). Chemosensitivity testing of human lung cancer cell lines using the MTT assay. *Br. J. Cancer*, **57**, 540.

COLES, S.P.C., DOWNES, H.F. & SLOVAK, M.L. (1989). Effect of calcium antagonists on the chemosensitivity of two multidrug resistant cell lines which do not overexpress P-glycoprotein. *Br. J. Cancer*, **59**, 42.

COLEY, H.M., TWENTYMAN, P.R. & WORKMAN, P. (1989*a*). Identification of anthracyclines and related agents that retain preferential activity over adriamycin in multidrug resistant cell lines and further resistance modification by verapamil and cyclosporin A. *Cancer Chemother. Pharmacol.*, **24**, 284.

COLEY, H.M., TWENTYMAN, P.R. & WORKMAN, P. (1989*b*). Improved cellular accumulation is characteristic of anthracyclines which retain high activity in multidrug resistant cell lines, alone or in combination with verapamil or cyclosporin A. *Biochem. Pharmacol.*, **38**, 4467.

COLEY, H.M., TWENTYMAN, P.R. & WORKMAN, P. (1990). 9-Alkyl, morpholinyl anthracyclines in the circumvention of multidrug resistance. *Eur. J. Cancer*, **26**, 665.

COLEY, H.M., TWENTYMAN, P.R., WORKMAN, P. & REEVE, J.G. (1989*c*). Anthracycline cross-resistance and drug accumulation in a multidrug resistant human lung cancer line which does not express P-170 glycoprotein. *Br. J. Cancer*, **60**, 496.

CROOP, J.M., GUILD, B.C., GROS, P., MULLIGAN, R. & HOUSMAN, D.E. (1987). Genetics of MDR: relationship of a cloned gene to the complete MDR phenotype. *Cancer Res.*, **47**, 5982.

DANKS, M.K., SCHMIDT, C.A., CIRTAIN, M.C., SUTTLE, D.P. & BECK, W.T. (1988). Altered catalytic activity of and DNA cleavage by DNA topoisomerase II from human leukaemic cells selected for resistance to VM-26. *Biochemistry*, **27**, 8861.

DANKS, M.K., YALOWICH, J.C. & BECK, W.T. (1987). Atypical multidrug resistance in a human leukaemic cell line selected for resistance to teniposide. *Cancer Res.*, **47**, 1297.

DANO, K. (1973). Active outward transport of daunomycin in resistant Ehrlich ascites tumour cells. *Biochem. Biophys. Acta*, **323**, 466.

ENDICOTT, J.A. & LING, V. (1989). The biochemistry of P-glycoprotein-mediated multidrug resistance. *Ann. Rev. Biochem.*, **58**, 137.

FOJO, A., AKIYAMA, S., GOTTESMAN, M.M. & PASTAN, I. (1985). Reduced drug accumulation in multiply drug resistant human KB carcinoma cell lines. *Cancer Res.*, **45**, 3002.

GOLDSTEIN, L.J., GASKI, H., FOJO, A. & 11 others (1989). Expression of a multidrug resistance gene in human cancers. *J. Natl Cancer Inst.*, **81**, 116.

HABER, M., NORRIS, M.D., KAVALLARIS, M. & 4 others (1990). Atypical multidrug resistance in a therapy induced drug-resistant human leukaemia cell line (LALW-2): resistance in vinca alkaloids independent of P-glycoprotein. *Cancer Res.*, **49**, 5281.

HARKER, W.G., SLADE, L.S., DALTON, W.S., MELTZER, P.S. & TRENT, J.M. (1989). Multidrug resistance in mitoxantrone-selected HL-60 leukaemia cells in the absence of P-glycoprotein over-expression. *Cancer Res.*, **49**, 4542.

INABA, M. & JOHNSON, R.K. (1978). Uptake and retention of adriamycin and daunorubicin by sensitive and anthracycline resistant sublines of P388 leukaemia. *Biochem. Pharmacol.*, **27**, 2121.

INABA, M., KOBAYASHI, H., SAKURAI, Y. & JOHNSON, R.K. (1979). Active efflux of daunorubicin and adriamycin in sensitive and resistant sublines of P388 leukaemia. *Cancer Res.*, **39**, 2200.

JULIANO, R. & LING, V. (1976). A surface glycoprotein modulating drug permeability in Chinese hamster ovary cell mutants. *Biochim. Biophys. Acta.*, **455**, 152.

KARTNER, N., EVERDEN-PORELLE, D., BRADLEY, G. & LING, V. (1985). Detection of P-glycoprotein in multidrug resistant cell lines by monoclonal antibodies. *Nature*, **316**, 820.

KARTNER, N., RIORDAN, J.R. & LING, V. (1983). Cell surface P-glycoprotein associated with multidrug resistance in mammalian cell lines. *Science*, **221**, 1285.

KESSEL, D. (1985). Promotion of daunorubicin uptake and toxicity by the calcium antagonist tiapamil and its analogs. *Cancer Treat. Rep.*, **69**, 673.

KESSEL, D. & CORBETT, T. (1985). Correlations between anthracycline resistance, drug accumulation and membrane glycoprotein patterns in solid tumours of mice. *Cancer Letts.*, **28**, 187.

KESSEL, D. & WILBERDING, C. (1985). Anthracycline resistance in P388 murine leukaemia and its circumvention by calcium antagonists. *Cancer Res.*, **45**, 1687.

LAI, S.L., GOLDSTEIN, L.J., GOTTESMAN, M.M. & 7 others (1989). MDR 1 expression in lung cancer. *J. Natl Cancer Inst.*, **81**, 1144.

MARQUARDT, D., MCCRONE, S. & CENTER, M.S. (1990). Mechanisms of multidrug resistance in HL60 cells: detection of resistance-associated proteins with antibodies against synthetic peptides that correspond to the deduced sequence of P-glycoprotein. *Cancer Res.*, **50**, 1426.

MARSH, W. & CENTER, M.S. (1987). Adriamycin resistance in HL60 cells and accompanying modifications of a membrane protein contained in drug-sensitive cells. *Cancer Res.*, **47**, 5080.

MARSH, W., SICHERI, D. & CENTER, M.S. (1986). Isolation and characterisation of adriamycin resistant HL-60 cells which are not defective in the initial intracellular accumulation of drug. *Cancer Res.*, **46**, 4053.

MIRSKI, S.E.L., GERLACH, J.H. & COLE, S.P.C. (1987). Multidrug resistance in a human small cell lung cancer cell line selected in adriamycin. *Cancer Res.*, **47**, 2594.

RAMU, A., FUKS, Z., GATT, S. & GLAUBIGER, D. (1984). Reversal of acquired resistance to doxorubicin in P388 murine leukaemia cells by perhexiline maleate. *Cancer Res.*, **44**, 144.

REEVE, J.G., RABBITTS, P.H. & TWENTYMAN, P.R. (1989*a*). Amplification and expression of mdr-1 gene in a multidrug resistant variant of small cell lung cancer line NCI-H69. *Br. J. Cancer*, **60**, 339.

REEVE, J.G., RABBITTS, P.H. & TWENTYMAN, P.R. (1990). Non-P-glycoprotein-mediated multidrug resistance with reduced EGF receptor expression in a human large cell lung cancer cell line. *Br. J. Cancer*, **61**, 851.

REEVE, J.G., WRIGHT, K.A., RABBITTS, P.H., TWENTYMAN, P.R. & KOCH, G. (1989*b*). Collateral resistance to verapamil in multidrug-resistant mouse tumour cells. *J. Natl Cancer Inst.*, **81**, 1588.

SCHWARTZ, H.S. (1973). A fluorimetric assay for daunorubicin and adriamycin in animal tissues. *Biochem. Med.*, **7**, 396.

SCOTT, C.A., WESTMACOTT, D., BROADHURST, M.J., THOMAS, G.I. & HALL, M.J. (1986). 9-alkyl anthracyclines. Absence of cross-resistance to adriamyin in human and murine cell cultures. *Br. J. Cancer*, **53**, 595.

SLOVAK, M.L., HOELTGE, G.A., DALTON, W.S. & TRENT, J.M. (1988). Pharmacological and biological evidence for differing mechanisms of doxorubicin resistance in two human tumour cell lines. *Cancer Res.*, **48**, 2793.

TSURUO, T., IIDA, H., KITATANI, V., YOKOTA, K., TSUKAGOSHI, S. & SAKURAI, Y. (1984). Effect of quinidine and related compounds on cytotoxicity and cellular accumulation of vincristine and adriamycin in drug-resistant tumour cell lines. *Cancer Res.*, **44**, 4303.

TSURUO, T., IIDA, H., TSUKAGOSHI, S. & SAKURAI, Y. (1983). Potentiation of vincristine and adriamycin effects in human haemopoietic tumour cell lines by calcium antagonists and calmodulin inhibitors. *Cancer Res.*, **43**, 2267.

TWENTYMAN, P.R., FOX, N.E., WRIGHT, K.A. & BLEEHEN, N.M. (1986*b*). Derivation and preliminary characterisation of adriamycin resistant lines of human lung cancer cell lines. *Br. J. Cancer*, **53**, 529.

TWENTYMAN, P.R., FOX, N.E., WRIGHT, K.A. & 4 others (1986*a*). The *in vitro* effects and cross-resistance patterns of some novel anthracyclines. *Br. J. Cancer*, **53**, 585.

TWENTYMAN, P.R. & LUSCOMBE, M. (1987). A study of some variables in a tetrazolium dye based assay for cell growth and chemosensitivity. *Br. J. Cancer*, **56**, 279.

ZIJISTRA, J.D., DE VRIES, E.G.E. & MULDER, N.H. (1987). Multifactorial drug resistance in an adriamycin-resistant human small cell lung carcinoma cell line. *Cancer Res.*, **47**, 1780.